# Genome sequence of the soil bacterium *Corynebacterium callunae* type strain DSM 20147^T^

**DOI:** 10.1186/1944-3277-10-5

**Published:** 2015-01-21

**Authors:** Marcus Persicke, Andreas Albersmeier, Hanna Bednarz, Karsten Niehaus, Jörn Kalinowski, Christian Rückert

**Affiliations:** 1 Technology Platform Genomics, CeBiTec, Bielefeld University, Bielefeld, Germany; 2 Proteome and Metabolome Research, Bielefeld University, Bielefeld, Germany

**Keywords:** Aerobic, Non-motile, Gram-positive, Non-spore forming, Glutamic acid producing

## Abstract

*Corynebacterium callunae* DSM 20147^T^ is a member of the genus *Corynebacterium* which contains Gram-positive and non-spore forming bacteria with a high G + C content. *C. callunae* was isolated during a screening for l-glutamic acid producing bacteria and belongs to the aerobic and non-haemolytic corynebacteria. As this is a type strain in a subgroup of industrial relevant bacteria for many of which there are also complete genome sequence available, knowledge of the complete genome sequence might enable genome comparisons to identify production relevant genetic loci. This project, describing the 2.84 Mbp long chromosome and the two plasmids, pCC1 (4.11 kbp) and pCC2 (85.02 kbp), with their 2,647 protein-coding and 82 RNA genes, will aid the *Genomic Encyclopedia of Bacteria and Archaea* project.

## Introduction

Strain DSM 20147^T^ is the type strain in a subgroup of industrial relevant bacteria originally isolated during a screening for l-glutamic acid producing microorganisms and was classified to belong to the genus *Corynebacterium*[1]. This genus is comprised of Gram-positive bacteria with a high G + C content. It currently contains 126 validly published members (species and subspecies), 4 of which are synonyms of other species within the genus, 27 that were later reclassified as members of 7 other genera, and 1 member abolished in erratum [2-11]. The remaining 93 were isolated from diverse backgrounds like soil, sea, or ripening cheese, but also from human clinical samples and animals.

Within this diverse genus, *C. callunae* has been found to be a producer of l-glutamic acid, like one of the most prominent representatives of the corynebacteria, *C. glutamicum*[1]. The biological context of this species is, unfortunately, basically unknown as it was first described in a patent application [1] that does neither mention the geographic location nor the exact habitat of the strain. Based on the name and the habitats of its close relatives *C. glutamicum*, *C. deserti*, and *C. efficiens*, the most likely habitat of *C. callunae* is soil around heather plants. But while the biotechnological uses and capabilities of this subgroup within the genus *Corynebacterium* has been studied in detail, especially for *C. glutamicum*, the ability of all these strains to secrete considerable amounts of l-glutamic acid is still not well understood in the context of the environment.

*C. callunae* DSM 20147^T^ harbors two cryptic plasmids: pCC1 (4,109 bp) which encodes a Rep protein that shows similarity to the corynebacterial plasmid pAG3 and pBL1, and pCC2 (85,023 bp) the Rep protein of which has possible orthologs in many other corynebacteria. Aside from this, DSM 20147^T^ is an alkaline-tolerant bacterium, which grows well at pH 5.0 - 9.0 (optimum pH 6–8) [1]. Here we present a summary classification and a set of features for *C. callunae* DSM 20147^T^, together with the description of the genomic sequencing and annotation.

## Organism information

### Classification and features

A representative genomic 16S rRNA sequence of *C. callunae* DSM 20147^T^ was compared to the Ribosomal Database Project database [12] confirming the initial taxonomic classification. *C. callunae* shows highest similarity to *C. glutamicum* and *C. deserti* (97%, respectively).

**Figure 1:**
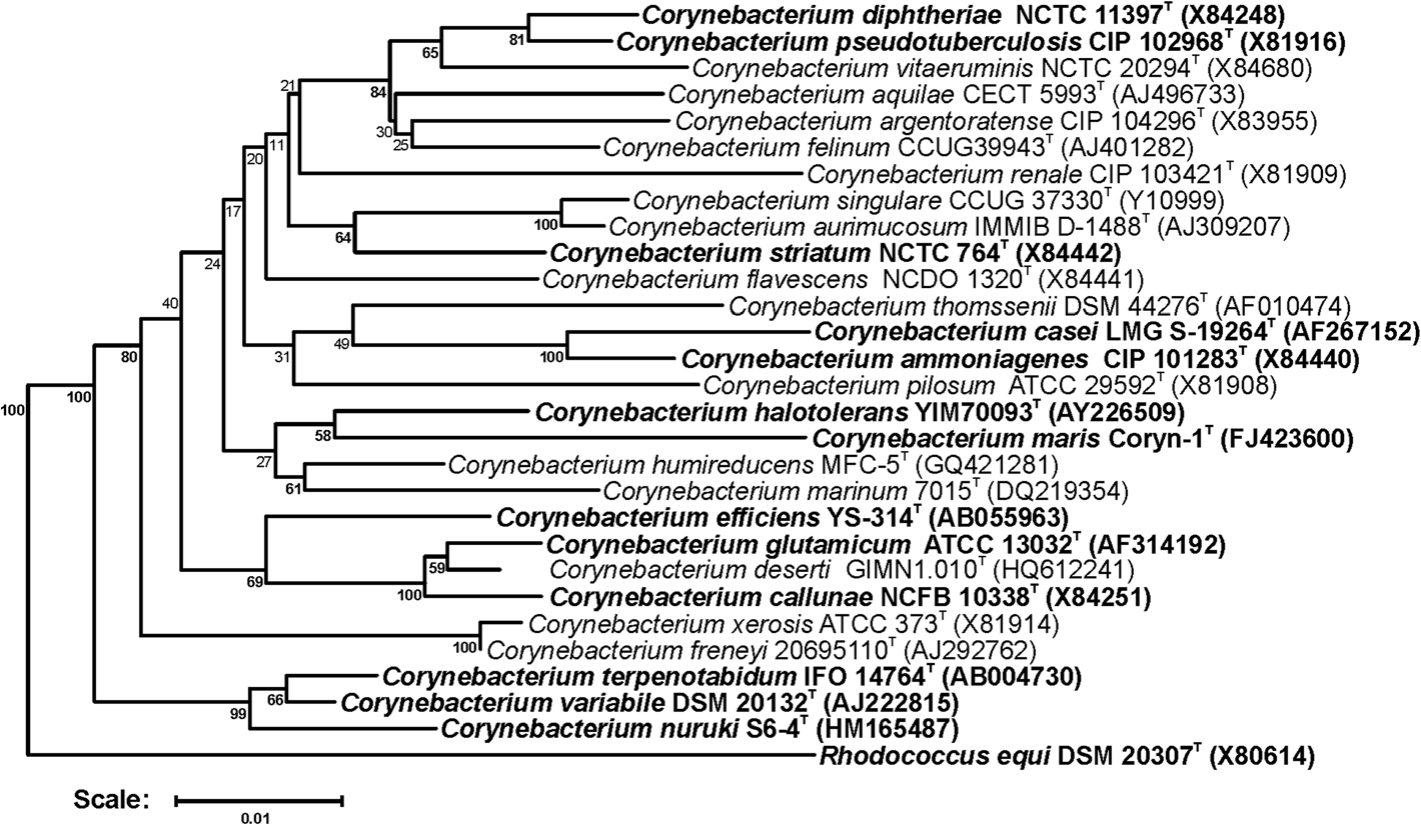
**Phylogenetic tree highlighting the position of *C. callunae *relative to type strains of other species within the genus *Corynebacterium*.** Species with at least one publicly available genome sequence (not necessarily the type strain) are highlighted in **bold face**. The tree is based on sequences aligned by the RDP aligner and utilizes the Jukes-Cantor corrected distance model to construct a distance matrix based on alignment model positions without alignment inserts, using a minimum comparable position of 200. The tree is built with RDP Tree Builder, which utilizes Weighbor [13] with an alphabet size of 4 and length size of 1000. The building of the tree also involves a bootstrapping process repeated 100 times to generate a majority consensus tree [14]*Rhodococcus equi* (X80614) was used as an outgroup.

Figure 1 shows the phylogenetic neighborhood of *C. callunae* in a 16S rRNA based tree. *C. callunae* forms a subgroup containing furthermore the species *C. glutamicum* ATCC 13032^T^, *C. deserti* GIMN1.010^T^, and *C. efficiens* YS-314^T^.

**Figure 2:**
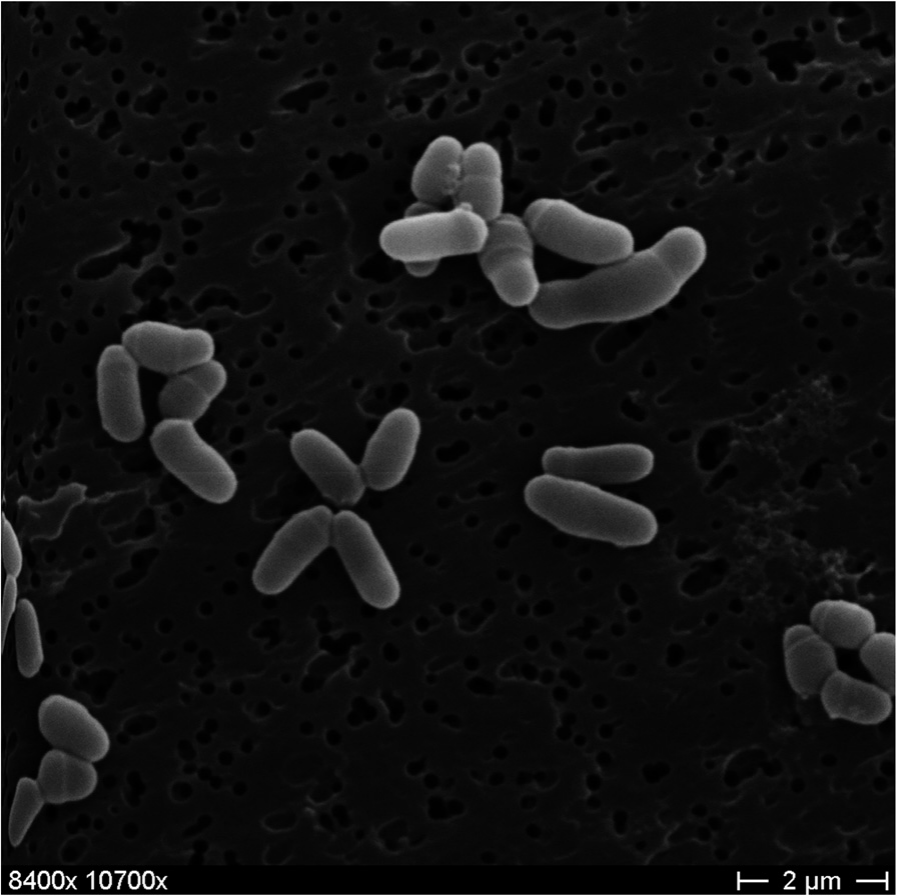
**Scanning electron micrograph of *C. callunae *** **DSM 20147**^**T**^**.**

*C. callunae* DSM 20147^T^ is a Gram-positive rod shaped bacterium, which is 1–2 μm long and 0.4-0.6 μm wide (Figure 2). It is described to be non-motile [1], which coincides with a complete lack of genes associated with ‘cell motility’ (functional category N in COGs table). Growth of DSM 20147^T^ was shown at temperatures between 25–37°C with optimal l-glutamic acid production between 25–35°C [1]. Carbon sources utilized by strain DSM 20147^T^ include dextrose, fructose, galactose, inulin, inositol, maltose, mannitol, mannose, raffinose, salicin, sucrose and trehalose [1]. DSM 20147^T^ tested positive for citrate, catalase and urease, but shows no nitrate reduction activity [1]. Details on the chemotaxonomy are largely missing, but can be inferred from the close relatives *C. glutamicum*, *C. efficiens*, and *C. deserti*[3]. Based on these relatives, *meso*-diaminopimelic acid is expected to be the major diamino acid of the cell wall, with arabinose and galactose as the main sugars (chemotype IV). Short-chain mycolic acids (32 ± 36 carbon atoms) are also certain to be present, as all necessary genes were found to be present. The major cellular fatty acids are expected to be hexadecanoic acid (C_16:0_, 40-50%) and octadecenoic acid (C_18:1_*ω*9*c*, 40-50%) with small amounts of octadecanoic acid (C_18:0_, ~1%) and possible others. MK-9(H_2_) is thought to be the major menaquinone, although MK-8(H_2_) might also be present in significant amounts. Phosphatidylinositol, diphosphatidylglycerol, and phosphatidylglycerol as well as their glycosides are expected to be the main components of the polar lipids (Table 1).

**Table 1:** **Classification and general features of *C. callunae *** **DSM 20147**^**T **^**according to the MIGS recommendations**[15]

**MIGS ID**	**Property**	**Term**	**Evidence code**^**a)**^
	Current classification	Domain *Bacteria*	TAS [16]
		Phylum ‘*Actinobacteria*’	TAS [17]
		Class *Actinobacteria*	TAS [18,19]
		Order *Actinomycetales*	TAS [18,20-22]
		Family *Corynebacteriaceae*	TAS [18,20,22,23]
		Genus *Corynebacterium*	TAS [24,25]
		Species *Corynebacterium callunae*	TAS [1,22,26]
		Type-strain DSM 20147	TAS [1,22,26]
	Gram stain	Positive	TAS [1]
	Cell shape	Rod-shaped	TAS [1]
	Motility	Non-motile	TAS [1]
	Sporulation	Non-sporulating	TAS [1]
	Temperature range	Mesophile	TAS [1]
	pH range	5 - 9; optimum 6 - 8	TAS [1]
	Salinity	Not reported	TAS [1]
MIGS-22	Oxygen requirement	Aerobe	TAS [1]
	Carbon source	Dextrose, fructose, galactose, inulin, inositol, maltose, mannitol, mannose, raffinose, salicin, sucrose and trehalose	TAS [1]
	Energy metabolism	Chemoorganoheterotrophic	NAS
	Terminal electron acceptor	Oxygen	NAS
MIGS-6	Habitat	Not reported	TAS [1]
MIGS-15	Biotic relationship	Free living	NAS
MIGS-14	Pathogenicity	Non-pathogenic	NAS
	Biosafety level	1	NAS
MIGS-23.1	Isolation	Not reported	TAS [1]
MIGS-4	Geographic location	Not reported	TAS [1]
MIGS-5	Sample collection time	Not reported	TAS [1]

^a)^Evidence codes - TAS: Traceable Author Statement (i.e., a direct report exists in the literature); NAS: Non-traceable Author Statement (i.e., not directly observed for the living, isolated sample, but based on a generally accepted property for the species, or anecdotal evidence). These evidence codes are from the Gene Ontology project [27].

## Genome sequencing and annotation

### Genome project history

Due to its phylogenetic position in the near neighborhood of industrial relevant species of the genus *Corynebacterium*, *C. callunae* was selected for sequencing as part of a project to define production relevant loci in corynebacteria. While not being part of the GEBA project, sequencing of the type strain will nonetheless aid the GEBA effort. The genome project is deposited in the Genomes OnLine Database [28] and the complete genome sequence is deposited in GenBank. Sequencing, finishing and annotation were performed at the CeBiTec. A summary of the project information is shown in Table 2.

**Table 2:** Genome sequencing project information

**MIGS ID**	**Property**	**Term**
MIGS-31	Finishing quality	Finished
MIGS-28	Libraries used	Nextera DNA Sample Prep Kit, Nextera Mate Pair Sample Prep Kit
MIGS-29	Sequencing platforms	Illumina MiSeq
MIGS-31.2	Sequencing coverage	99.51×
MIGS-30	Assemblers	Newbler version 2.8
MIGS-32	Gene calling method	GeneMark, Glimmer
	Locus Tag	H924
	Genbank ID	CP004354, CP004355, CP004356
	GenBank Date of Release	March 6, 2013
	GOLD ID	Gc0042965
	BIOPROJECT ID	190670
	Project relevance	Industrial, GEBA
MIGS-13	Source material identifier	DSM 20147

### Growth conditions and DNA isolation

*C. callunae* DSM 20147^T^ was grown aerobically in CASO bouillon (Carl Roth GmbH, Karlsruhe, Germany) at 30°C. DNA was isolated from ~ 10^8^ cells using the protocol described by Tauch *et al*. [29].

### Genome sequencing and assembly

Two libraries were prepared: a WGS library using the Illumina-Compatible Nextera DNA Sample Prep Kit (Epicentre, WI, U.S.A) and a 6 k MatePair library using the Nextera Mate Pair Sample Preparation Kit, both according to the manufacturer's protocol. Both libraries were sequenced in a 2× 250 bp paired read run on the MiSeq platform, yielding 1,747,266 total reads, providing 99.51× coverage of the genome. Reads were assembled using the Newbler assembler v2.8 (Roche). The initial Newbler assembly consisted of 29 contigs in four scaffolds. Analysis of the four scaffolds revealed two to be an extrachromosomal element (plasmid pCC1 and pCC2), one to make up the chromosome and the remaining one containing the seven copies of the RRN operon.

The Phred/Phrap/Consed software package [30-33] was used for sequence assembly and quality assessment in the subsequent finishing process, gaps between contigs were closed by manual editing in Consed (for repetitive elements).

### Genome annotation

Gene prediction and annotation were done using the PGAP pipeline [34]. Genes were identified using GeneMark [35], GLIMMER [36], and Prodigal [37]. For annotation, BLAST searches against the NCBI Protein Clusters Database [38] are performed and the annotation is enriched by searches against the Conserved Domain Database [39] and subsequent assignment of coding sequences to COGs. Non-coding genes and miscellaneous features were predicted using tRNAscan-SE [40], Infernal [41], RNAMMer [42], Rfam [43], TMHMM [44], and SignalP [45].

## Genome properties

**Figure 3:**
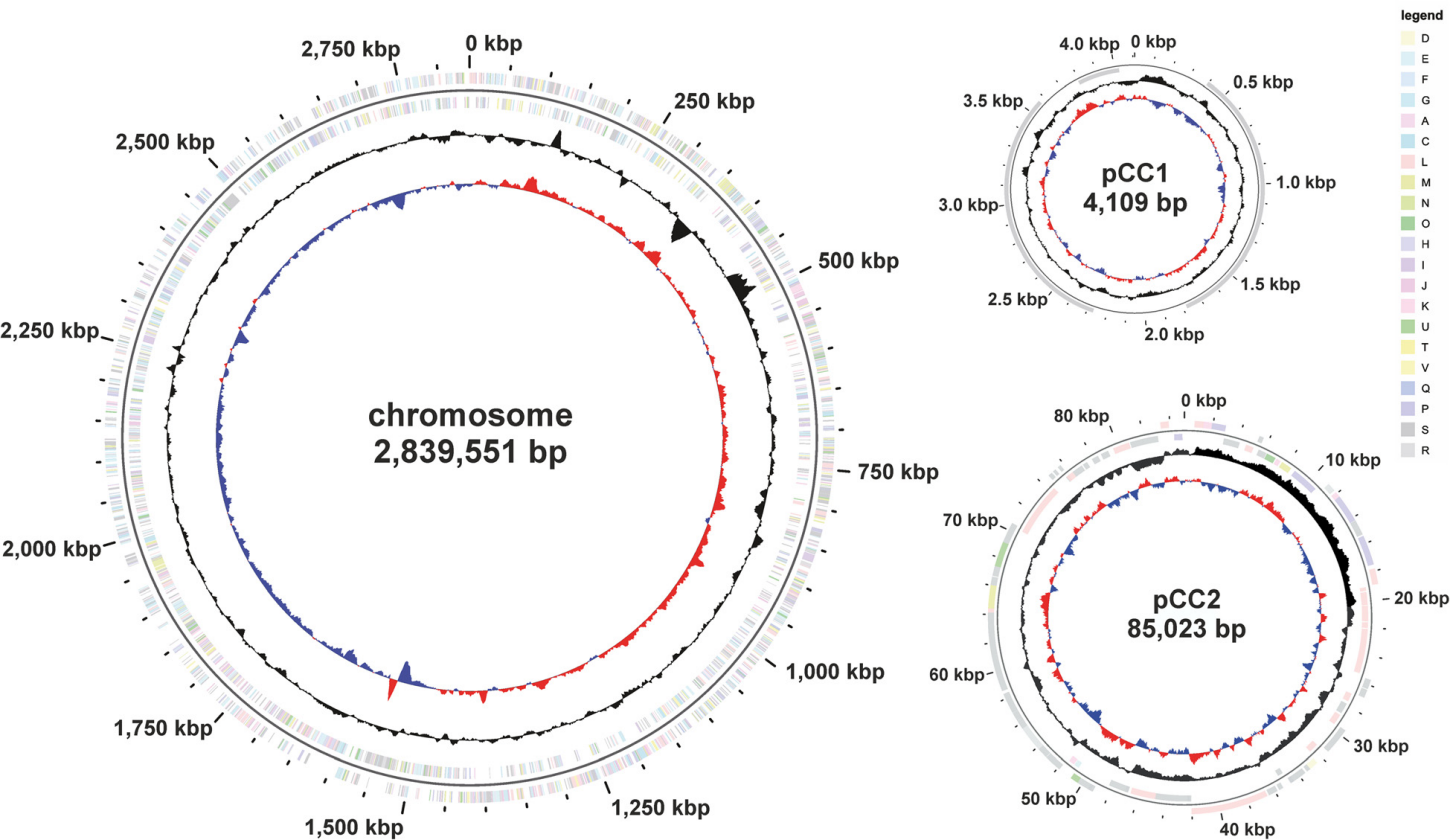
**Graphical map of the chromosome and the two plasmids pCC1 and pCC2 (not drawn to scale).** From the outside in: Genes on forward strand (color by COG categories), Genes on reverse strand (color by COG categories), GC content, GC skew.

The genome (on the scale of 2,928,683 bp) includes one circular chromosome of 2,839,5514 bp (52.39% G + C content) and two plasmids of 4,109 bp (54.42% G + C content) and 85,023 bp (54.38% G + C content, [Figure 3]). For chromosome and plasmids, a total of 2,729 genes were predicted, 2,647 of which are protein coding genes. 2,085 (76.40%) of the protein coding genes were assigned to a putative function, the remaining were annotated as hypothetical proteins. 1,937 protein coding genes belong to 314 paralogous families in this genome corresponding to a gene content redundancy of 41.52%. The properties and the statistics of the genome are summarized in [Tables 3, 4 and 5].

**Table 3:** Summary of genome: one chromosome and two plasmids

**Label**	**Size (Mb)**	**Topology**	**INSDC identifier**
Chromosome	2.840	circular	CP004354
Plasmid pCC1	0.004	circular	CP004355
Plasmid pCC2	0.085	circular	CP004356

**Table 4:** Genome statistics

**Attribute**	**Value**	**% of total**^**a**^
Genome size (bp)	2,928,683	100.00
DNA coding (bp)	2,678,511	91.46
DNA G + C (bp)	1,536,292	52.46
DNA scaffolds	3	
Total genes	2,729	100.00
Protein coding genes	2,647	97.00
RNA genes	82	3.00
Pseudo genes	61	2.24
Genes in internal clusters	1,937	64.05
Genes with function prediction	2,085	76.40
Genes assigned to COGs	1,748	41.52
Genes with Pfam domains	2,125	5.06
Genes with signal peptides	158	5.79
Genes with transmembrane helices	673	24.66
CRISPR repeats	0	

^a)^The total is based on either the size of the genome in base pairs or the total number of total genes in the annotated genome.

**Table 5:** Number of genes associated with the general COG functional categories

**Code**	**Value**	**% age**	**Description**
J	148	5.59	Translation, ribosomal structure and biogenesis
A	1	0.04	RNA processing and modification
K	174	6.57	Transcription
L	192	7.25	Replication, recombination and repair
B	0	0.00	Chromatin structure and dynamics
D	20	0.76	Cell cycle control, cell division, chromosome partitioning
Y	0	0.00	Nuclear structure
V	41	1.55	Defense mechanisms
T	66	2.49	Signal transduction mechanisms
M	116	4.38	Cell wall/membrane biogenesis
N	1	0.04	Cell motility
Z	0	0.00	Cytoskeleton
W	1	0.04	Extracellular structures
U	28	1.06	Intracellular trafficking and secretion, and vesicular transport
O	76	2.87	Posttranslational modification, protein turnover, chaperones
C	115	4.34	Energy production and conversion
G	173	6.54	Carbohydrate transport and metabolism
E	244	9.22	Amino acid transport and metabolism
F	74	2.80	Nucleotide transport and metabolism
H	107	4.04	Coenzyme transport and metabolism
I	57	2.23	Lipid transport and metabolism
P	182	6.88	Inorganic ion transport and metabolism
Q	53	2.00	Secondary metabolites biosynthesis, transport and catabolism
R	315	11.90	General function prediction only
S	170	6.42	Function unknown
-	629	23.76	Not in COGs

### Insights from the genome sequence

**Figure 4:**
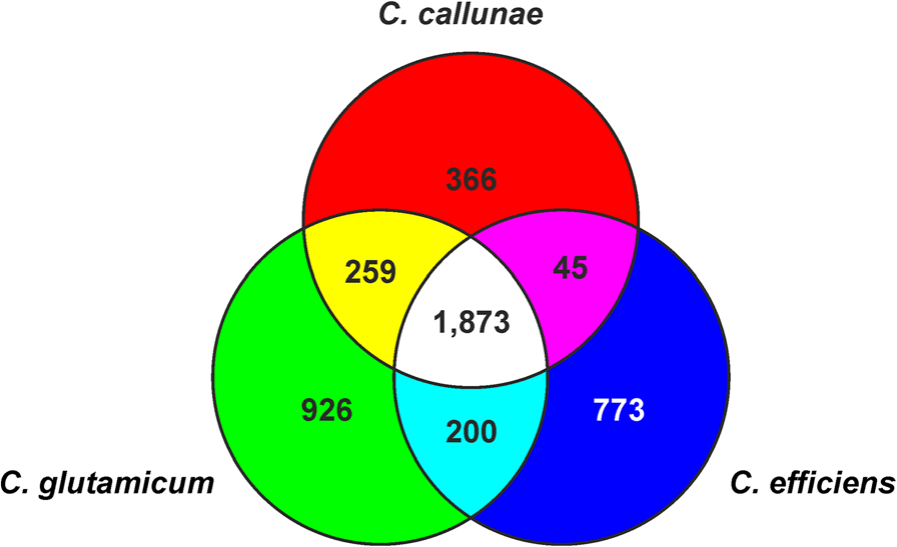
**Venn diagram depicting the number of genes shared between *C. callunae*, *C. glutamicum*, and *C. efficiens*.** EDGAR [47] was used to determine the core genomes shared between respectively singletons unique to the three species.

The complete genome sequence of *C. callunae* was already mined for biotechnological purposes to define the core genome of the *C. glutamicum* - *C. efficiens *- *C. callunae* subgroup to define the chassis genome for *C. glutamicum*[46]. Comparison of the three genomes using EDGAR [47] reveals that the core genome of this group comprises just 1,873 genes and the number of genes that are found only in *C. callunae* is also relatively small (366), especially when compared to number of singletons found in the other two (926 and 773 in *C. glutamicum* and *C. efficiens*, respectively; Figure 4). As *C. callunae* was shown to produce l-glutamate in an amount comparable to *C. glutamicum*, *C. callunae* might be considered as a potential candidate for future genome reduction efforts since the chromosome is already considerably smaller than that of *C. glutamicum* and *C. efficiens* (2.84 Mbp versus 3.21 Mbp and 3.15 Mbp, respectively). This future approach is aided by the observation that many of the singletons are clustered in just three regions (I: H924_2045-H924_02230, 37 genes, 25.2 kbp; II: H924_03630-H924_03880, 50 genes 52.5 kbp; III: H924_07070-H924_07380, 61 genes, 48.2 kbp) which constitutes ~ 4.4% of the genome size. As at least region II and region III are likely prophages, loss of these regions should be neutral or even beneficial, as demonstrated for *C. glutamicum*[48].

One central prerequisite for future rational strain development is the genetic accessibility of the prospective strain. Knowledge of the complete genome sequence of *C. callunae* helps to overcome at least two of the main obstacles: the construction of plasmids usable as vectors and removal of elements that hinder DNA transfer. For the former, the knowledge of the sequences of the two plasmids pCC1 and pCC2 allows use of plasmid-tagging approaches via a counter-selectable marker [49] to cure them, should conventional approaches like heat-shock curing fail. Once cured, the sequence of the plasmids help to identify the minimal gene set necessary for replication to build shuttle vectors, as demonstrated for pCC1 [50]. For the latter, the genome sequence helps to identify restriction-modification systems. A preliminary analysis revealed the presence of at least 4 such systems, one of which is located in the potential prophage region II. Removal of such systems has been shown to significantly enhance the stability of foreign DNA introduced and thus facilitating genetic engineering approaches [48].

## Conclusion

The complete genome sequence of *C. callunae* is the third genome sequence of the *C. glutamicum* - *C. deserti* - *C. efficiens* - *C. callunae* subgroup of L-glutamic acid producing corynebacteria within the genus *Corynebacterium*. Knowledge of the complete genome sequence has already contributed to identify the core genome of this group. With a size of 2.84 Mbp and an a total of 2,647 protein coding genes, the genome of *C. callunae* is by far the smallest within this group. Therefore, this bacterium might be an ideal choice for future development of a platform strain as the otherwise high degree of similarity of its genome content to the well studied *C. glutamicum* would allow an easy transfer of knowledge to the new host. Furthermore, knowledge of the complete genome sequence also facilitates the identification of possible targets to improve the accessibility to genetic engineering (like restriction-modification systems) and to enhance genome stability (like phages and transposases).

## Abbreviations

CeBiTec: Center for Biotechnology; GEBA: *Genomic Encyclopedia of Bacteria and Archaea.*

## Competing interests

The authors declare that they have no competing interests.

## Authors contributions

MP prepared and wrote the manuscript, AA performed library preparation and sequencing, HB and KN performed electron microscopy, JK coordinated the study, and CR assembled and analyzed the genome sequence.
